# Novel Silent Mutations in the *HIRA* Gene Associated with Litter Size in Sonid Sheep

**DOI:** 10.3390/ani15202936

**Published:** 2025-10-10

**Authors:** Chen Wang, Zhana Naren, He Bu, Ming Cang, Guifang Cao, Buhe Nashun, Bin Tong

**Affiliations:** 1 The State Key Laboratory of Reproductive Regulation and Breeding of Grassland Livestock, School of Life Sciences, Inner Mongolia University, Hohhot 010020, China; 2 Sonid Right Banner Sonid Sheep Breeding Technology Company, Xilingol 011207, China; 3 Sonid Left Banner Livestock Germplasm Development Company, Xilingol 011364, China

**Keywords:** association, *HIRA*, litter size, novel mutation, sonid sheep

## Abstract

Improving ovine reproductive efficiency is an important breeding goal that could substantially enhance economic viability in the sheep industry. The histone cell cycle regulator (*HIRA*) gene has been functionally validated as a reproductive regulator in model organisms such as *Drosophila* and murine species; however, research on the effects of *HIRA* on the prolificacy in sheep remains scarce. Thus, this study aimed to investigate the association between genetic variants of *HIRA* and litter size in sheep. In brief, we identified 15 novel exonic mutations in the Sonid sheep breed via direct sequencing. Notably, a linkage disequilibrium including the c.1521C>G, c.1572C>T, and c.1578G>A mutations on exon 14 of *HIRA* exhibited a significant association with litter size in Sonid sheep (*p* < 0.01). Meanwhile, it was predicted that mutations play a major role in enhancing the stability of the mRNA secondary structure by lowering the minimum free energy, and mutations were also thought to change the mRNA secondary structure of ovine *HIRA*. Therefore, these findings may provide potentially useful molecular markers for optimizing reproductive performance in Sonid sheep.

## Simple Summary

This study investigated the association between genetic variants of the *HIRA* gene and litter size in Sonid sheep. Through analysis, we identified fifteen novel mutations in the *HIRA* gene. Among these, the c.1521C>G, c.1572C>T, and c.1578G>A variants were associated with litter size, while c.1273G>A and c.1735A>G were predicted to alter protein structure. These variants could serve as valuable genetic markers for improving prolificacy in sheep breeding and highlight new variants for further functional research on the *HIRA* gene.

## 1. Introduction

Sheep is one of the most important livestock species globally, with a world inventory of over 1.3 billion head, contributing significantly to meat, wool, and milk production. China has the world’s largest sheep inventory, exceeding 170 million, with Inner Mongolia being a key production region. Sheep husbandry is a cornerstone of the agricultural economy and social fabric of Inner Mongolia [1,2]. Improving the reproductive efficiency of local breeds like Sonid sheep is a high-priority goal for enhancing food security and herders’ livelihoods in this region.

Sonid sheep is a local, indigenous, coarse wool breed found in Inner Mongolia, China, predominantly distributed across the Sonid grassland of the Mongolian Plateau under arid continental climatic conditions [3]. The meat of Sonid sheep is very popular among consumers in Inner Mongolia and even all of China, since it is palatable and recognized as a natural green food [2,3]. However, the production rate of Sonid sheep is constrained by seasonal estrus and low fertility (mean litter size is 1.03~1.13). Currently, the three primary genes recognized as influencing sheep prolificacy are bone morphogenetic protein receptor 1B (*BMPR1B*), bone morphogenetic protein 15 (*BMP15*), and growth differentiation factor 9 (*GDF9*). They all belong to the transforming growth factor β (TGF-β) superfamily, located on the 6, X, and 5 chromosomes of sheep, respectively. Mutations in these genes can influence the ovulation rate and litter size of ewes [3,4,5]. The *FecB* mutation of *BMPR1B*, which was first discovered in Booroola Merino sheep, is the main gene responsible for high prolificacy in sheep. These genes that influence the ovulation rate and litter size have been confirmed across various sheep populations worldwide. While mutations in *BMPR1B*, *BMP15*, and *GDF9* are major well-established genes in terms of prolificacy, their effects are breed-specific, and they do not account for all genetic variation in litter size. Therefore, investigating novel candidate genes, such as *HIRA*, which has been implicated in reproductive processes in other species, is crucial for improving prolificacy in Sonid sheep. To date, there have been no studies looking into the prolificacy of Sonid sheep. Thus, it is important to investigate breed-specific functional genes and markers associated with prolificacy in Sonid sheep.

Histone cell cycle regulator (HIRA) is a conserved histone chaperone for H3.3 [6], comprising 24 exons encoding 1020 amino acids that participate in chromatin regulatory processes, including transcriptional regulation, spermatid chromatin remodeling, and embryonic development [7,8]. As a member of an evolutionarily conserved protein family, HIRA orthologs have been identified across diverse organisms, including yeast, *Drosophila*, mice, human, and plants, playing essential roles in maintaining genomic integrity and cellular homeostasis [9,10]. In *Drosophila* models, HIRA deficiency leads to female sterility, in which maternal histones fail to incorporate into the male pronucleus during fertilization, thereby preventing paternal chromatin reorganization [11]. Vertebrate studies further revealed that HIRA mutations universally result in embryonic lethality [12]. Specifically, murine studies by Nashun et al. established that murine HIRA is indispensable for transcriptional regulation and DNA methylation in oocytes. Its absence disrupts H3.3/H4 histone replacement, thus inducing chromatin abnormalities and subsequent oocyte apoptosis [13]. In sheep, Zhou et al. demonstrated that two single-nucleotide mutations (SNPs), g.71874104G>A and g.71833755T>C in the *HIRA* gene, significantly influence litter size in Small-tail Han sheep [14], thus highlighting the possibility that the *HIRA* gene could be considered a candidate gene for reproductive performance in sheep.

Based on the established role of HIRA in reproduction and previous associations in other sheep breeds, we hypothesized that novel genetic variants in the *HIRA* gene are associated with litter size in Sonid sheep. Therefore, this study aims to achieve several objectives: (1) identify novel mutations in the *HIRA* gene of Sonid sheep; (2) analyze the association between the novel mutations of the *HIRA* gene and litter size in Sonid sheep; (3) assess the potential effects of these novel variants on the mRNA and protein characteristics and structure of the *HIRA* gene in sheep. Our findings may contribute to the development of effective marker-assisted selection strategies to enhance litter size in the Sonid sheep breed and offer new perspectives on the influence of the *HIRA* gene on reproductive traits in sheep.

## 2. Materials and Methods

### 2.1. Samples and Date

A total of 384 healthy, multiparous Sonid sheep ewes (aged 3–4 years, body weight 45–55 kg, with 1–2 previous lambings) were sampled from the Sonid Right Banner Sonid Sheep Breeding Technology Company and the Sonid Left Banner Livestock Germplasm Development Company in Inner Mongolia for association analysis. Sonid sheep is a breed characterized by seasonal estrus in autumn [1]. Based on lambing records from these companies from 2021 to 2023, we sampled 52 Sonid ewes that consistently produced twin litters over three consecutive years for genotyping while sampling 332 ewes that stably produced a single lamb. Blood samples of 384 sheep were collected in October 2023, then immediately placed on ice after collection, transported to the laboratory within 6 h, and stored at −80 °C until DNA extraction. To discover novel variants in the *HIRA* gene, a subset of 20 ewes (10 from the twin-lamb group and 10 from the single-lamb group) was randomly selected for sequencing. It is important to emphasize that these ewes were mated naturally without any selection of rams. All the sheep were maintained in similar environments with unrestricted access to food and water. Given the impact of the FecB mutation on the litter size in sheep, we initially screened the Sonid ewes for the presence of the FecB mutation, and our experimental animals did not exhibit any FecB mutation.

### 2.2. DNA Extraction and Sequencing

Twenty ewes were selected for the assessment of variants in the *HIRA* gene among Sonid sheep, consisting of ten ewes that consistently produced twin lambs and ten ewes that regularly had single lambs. DNA was extracted from 384 blood samples using the Tiangen Blood/Cell/Tissue Genomic DNA Extraction Kit (Tiangen, Beijing, China), in accordance with the instructions provided by the manufacturer. To avoid degradation, the quality and concentration of the extracted DNA were evaluated through agarose gel electrophoresis and with a Nanodrop One spectrophotometer (from Thermo, Waltham, MA, USA) after being stored at −20 °C.

For PCR amplification, 21 primer pairs were designed using Primer Premier 5.0 to target the exon regions of the ovine *HIRA* gene (Oar_rambouillet_v3.0, NCBI reference sequence: NC_056070.1), as outlined in Table 1 [2]. The total volume of each PCR is 50 μL, which includes 2 μL of the extracted DNA as a template, 1 μM of each primer, 25 μL of Ex Taq DNA polymerase (from Takara, Dalian, China), and 21 μL of ddH2O. The cycling conditions for the PCR were set as follows: an initial denaturation at 94 °C for 5 min, followed by 35 cycles of denaturation at 94 °C for 30 s, annealing for 30 s, and elongation at 72 °C for 1 min and 10 s, with a final extension at 72 °C for 10 min. The specific annealing temperatures for each PCR fragment are listed in Table 1. The quality and quantity of the PCR products were analyzed through 3.0% agarose gel electrophoresis. These products were subsequently sequenced by the Beijing Genomics Institute (BGI, Beijing, China).

### 2.3. SNP Genotyping Using iPLEX MassARRAY

Fifteen new variants were analyzed using the MassARRAY^®^ SNP genotyping system (Agena Bioscience, San Diego, CA, USA) in the experimental samples. The PCR and extension primers of the *HIRA* gene were designed from sequences encompassing each target mutation along with approximately 100 bases upstream and downstream, employing the Assay Design Suite v3.0 (http://agenabio.com/assay-design-suite-20-software, accessed on 10 October 2024) with default settings (Supplementary Table S1). The genotypes for each allele were assessed using the Sequenom MassARRAY iPLEX platform. The resulting data were processed with MassARRAY Typer 4.0 Analyzer software (Agena Bioscience, San Diego, CA, USA) [15].

### 2.4. Bioinformatic Analyses

**Figure 1:**
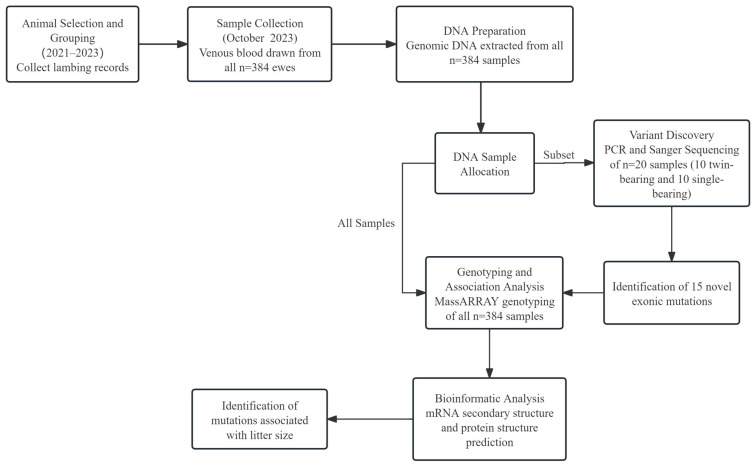
The experimental workflow of this study. The timeline illustrates the key steps, from animal selection based on lambing records (2021–2023) to bioinformatic analysis.

The fundamental characteristics of the predicted HIRA proteins were analyzed using ProtParam (https://web.expasy.org/protparam/, accessed on 2 December 2024). Potential transmembrane domains were identified through TMHMM (https://services.healthtech.dtu.dk/services/TMHMM-2.0/, accessed on 4 December 2024). The Simple Molecular Architecture Research Tool (SMART) (http://smart.embl-heidelberg.de/, accessed on 5 December 2024) was employed to predict the conserved domains of HIRA. N-glycosylation and phosphorylation sites were predicted with NetOGlyc 4.0 (https://services.healthtech.dtu.dk/services/NetOGlyc-4.0/, accessed on 12 December 2024) and NetPhos 3.1 (https://services.healthtech.dtu.dk/services/NetPhos-3.1/, accessed on 13 December 2024), respectively. The hydrophilicity and average flexibility index of the ovine HIRA amino acid sequences were evaluated using ProtScale (https://web.expasy.org/protscale/, accessed on 3 January 2025). RNAfold was utilized to forecast the mRNA secondary structure of the HIRA gene in sheep (http://rna.tbi.univie.ac.at//cgi-bin/RNAWebSuite/RNAfold.cgi, accessed on 4 January 2025). The secondary structure of ovine HIRA was determined with SOPMA (https://npsa.lyon.inserm.fr/cgi-bin/npsa_automat.pl?page=/NPSA/npsa_sopma.html, accessed on 4 January 2025), while its tertiary structure and template alignment were projected using SWISS-MODEL (https://swissmodel.expasy.org/, accessed on 7 January 2025). Multiple alignments and the construction of a molecular phylogenetic tree were carried out using Uniprot Align (https://www.uniprot.org/align, accessed on 10 January 2025) and MEGA-X (MEGA 11.0.13, https://www.megasoftware.net/docs, accessed on 15 January 2025). A schematic diagram of the experimental protocol is provided in Figure 1.

### 2.5. Statistical Analyses

Genotypic and allelic frequency calculations, along with Hardy–Weinberg equilibrium assessments, were performed for the Sonid sheep population. Population genetic metrics were derived using Nei’s methods [16], which included observed heterozygosity (H_o_), expected heterozygosity (H_e_), effective allele numbers (n_e_), and polymorphism information content (PIC). A chi-squared test was applied to compare the allelic frequencies of each variant. Linkage disequilibrium (LD), which encompassed D’ and *r*^2^ values, was evaluated using HAPLOVIEW version 4.2 [17]. The genetic effects of each SNP on the litter size of Sonid sheep were analyzed using a one-way analysis of variance (ANOVA) followed by Tukey’s multiple comparison test [18].

## 3. Results

### 3.1. Variant Discovery in HIRA

**Figure 2:**
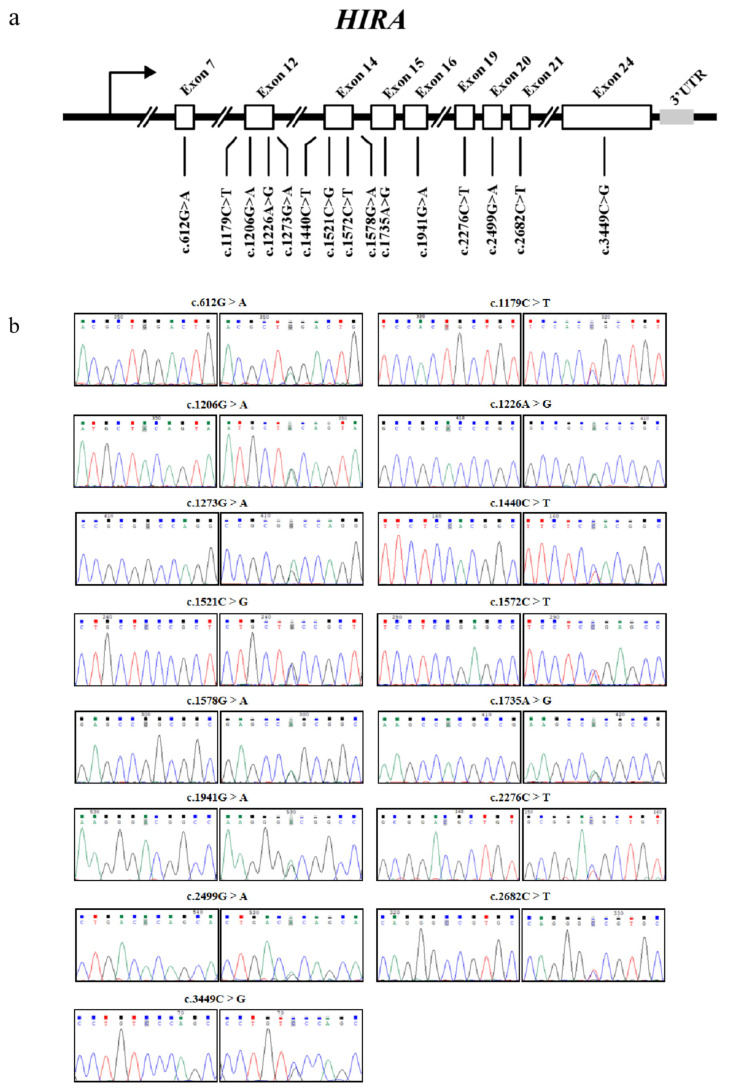
The identification of variants in the ovine *HIRA* gene. (**a**) The physical positions of the 15 variants identified in this study are illustrated. (**b**) The nucleotide substitutions corresponding to the 15 *HIRA* variants are presented. These variant locations were found according to chromosome X based on Oar_v3.0 (GenBank accession: NC_056070.1).

Through direct sequencing analysis, we identified 15 novel variants exclusively localized within the exonic regions of the *HIRA* gene, designated as the c.612G>A, c.1179C>T, c.1206G>A, c.1226A>G, c.1273G>A, c.1440C>T, c.1521C>G, c.1572C>T, c.1578G>A, c.1735A>G, c.1941G>A, c.2276C>T, c.2499G>A, c.2682C>T, and c.3449C>G mutations. The genomic distribution of these variants across the *HIRA* gene is schematically illustrated in Figure 2a,b.

**Figure 3:**
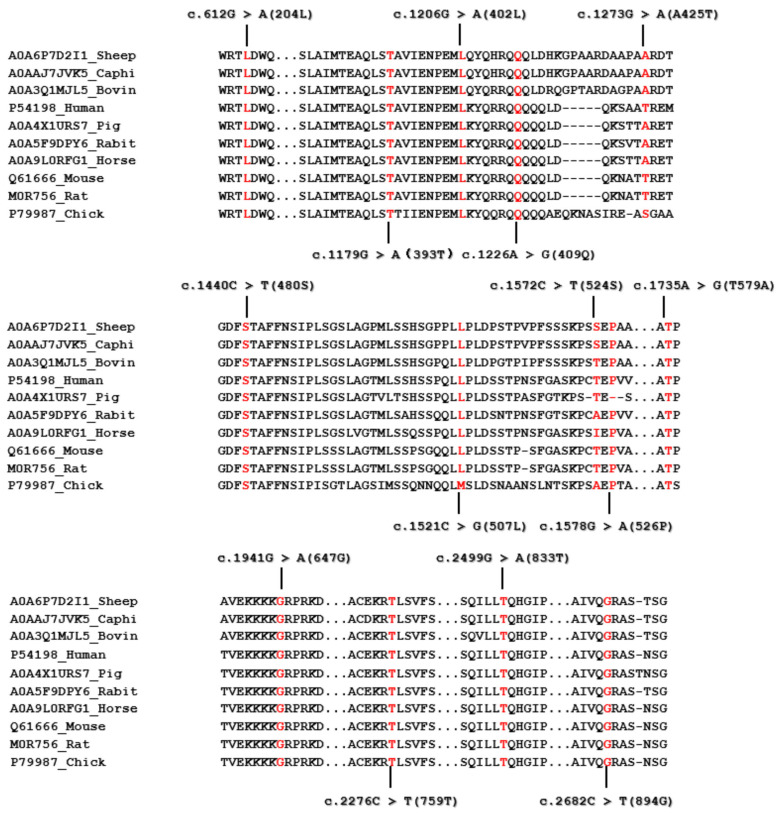
The alignment of the wild-type sequence of the HIRA protein across different species. The HIRA amino acid sequences of each species were sourced from the UniProt database.

Missense mutations were identified in the c.1273G>A (Ala425Thr) and 1735A>G (Thr579Ala) mutations of the *HIRA* gene. In contrast, all other variants showed synonymous changes. The HIRA protein aligns with a comparison of wild-type sequences from various species ranging from chicken to mammals, as illustrated in Figure 3.

### 3.2. Genetic Diversity Analysis

Allele frequencies, genotype distributions, and genetic indices (H_o_, H_e_, n_e_, PIC, and Hardy–Weinberg equilibria) for the identified variants in the Sonid sheep population are summarized in Table S2. Moderate polymorphism was detected at the c.1941G>A mutation, while all other variants displayed low polymorphism.

### 3.3. Linkage Disequilibrium Analysis of Novel Variants in HIRA

**Figure 4:**
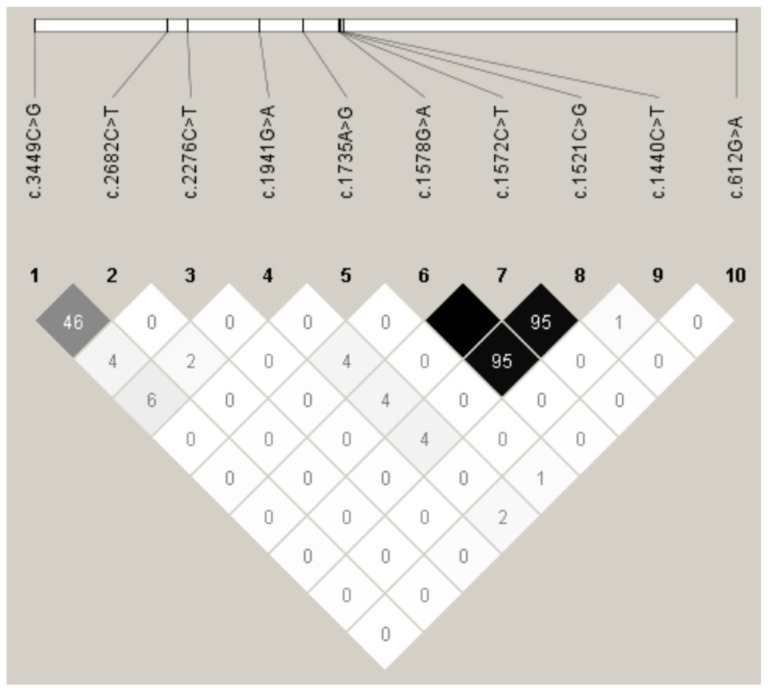
Linkage disequilibrium estimated among *HIRA* mutations in Sonid sheep population. Black represents *r*^2^ = 1, gray represents 0 < *r*^2^ < 1, and white represents *r*^2^ = 0.

Linkage disequilibrium (LD) analysis was performed for ten mutations that met the Hardy–Weinberg equilibrium (HWE) criteria. The remaining five identified variants were excluded from the LD analysis because they exhibited significant departure from HWE (*p* < 0.05), which can complicate the accurate estimation of haplotype frequencies and LD patterns. D’ and *r*^2^ values were also calculated for Sonid sheep. The analysis revealed near complete linkage disequilibrium (D’ = 1.0; *r*^2^ = 1.00) between c.1572C>T and c.1578G>A. Strong LD was also observed between c.1572C>T and c.1521C>G (D’ = 1.00; *r*^2^ = 0.953), as well as between c.1578G>A and c.1521C>G (D’ = 1.00; *r*^2^ = 0.954). Therefore, these mutations are designated as LD-Sonid for combined locus analysis. The remaining LD patterns are graphically represented in Figure 4.

### 3.4. Associations Between Genetic Variants and Litter Size

Association analyses were conducted in 384 Sonid ewes for seven variants. Among the fifteen identified mutations, five exhibited significant departures from HWE, and three were found in fewer than ten individuals. Consequently, these eight variants were excluded from the statistical computations. As the c.1521C>G, c.1572C>T, and c.1578G>A mutations were in LD (Figure 4), the association results for the combined LD-Sonid are represented by the c.1521C>G genotype in Table 2. The c.1521C>G, c.1572C>T, and c.1578G>A of LD-Sonid in exon 14 of *HIRA* were associated with litter size in Sonid sheep (*p* < 0.01). Detailed results are presented in Table 2.

### 3.5. Bioinformatic Analysis of Ovine HIRA

#### 3.5.1. Structural Characterization of Ovine HIRA Protein

**Figure 5:**
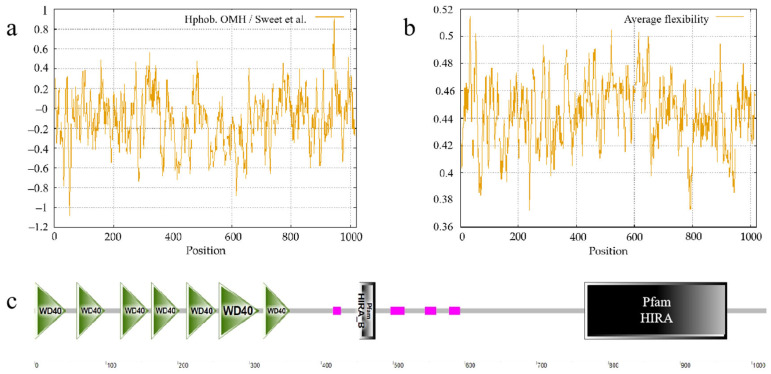
Prediction of features and structure for ovine HIRA protein. (**a**) Analysis of hydrophobicity of HIRA protein [19]. (**b**) Average flexibility index of HIRA protein. (**c**) Anticipated conserved domains of HIRA protein.

Hydrophobicity profiling identified peak hydrophobicity (0.904) at residue 945 and minimal hydrophobicity (−1.084) at residue 53 (Figure 5a). Flexibility analysis revealed the maximum average flexibility (0.514) at residue 34 and the minimum (0.372) at residue 238 (Figure 5b). Domain prediction via SMART identified seven WD40 repeats (1–44, 59–98, 120–159, 163–202, 212–254, 257–313, 319–356), four low-complexity regions (LCDs) (416–427, 496–516, 544–560, 578–593), a PFAM: HIRA_B domain (453–475), and a PFAM: HIRA domain (767–965) (Figure 5c). The variants c.1273G>A, c.1521C>G, and c.1735A>G are located within the LCD of HIRA. c.2499G>A and c.2682C>T are found within the PFAM: HIRA domain. Therefore, these mutations should be considered more significant than the other variants identified in this study.

#### 3.5.2. Physicochemical Properties of Ovine HIRA

**Figure 6:**
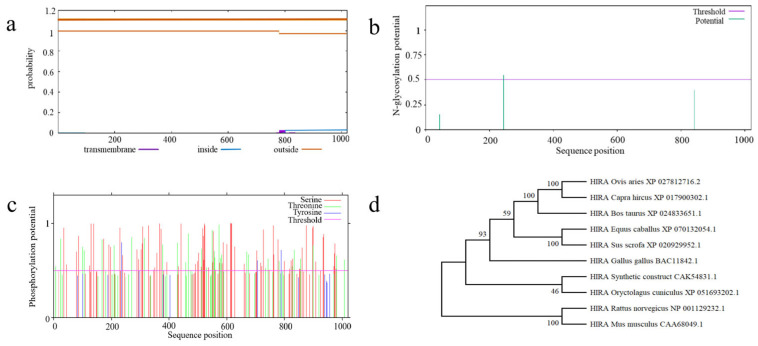
Amino acid sequence analysis of ovine *HIRA* along with its multiple sequence alignments. (**a**) Predicted transmembrane helices within HIRA protein. (**b**) Anticipated N-glycosylation sites of HIRA protein. (**c**) Forecasted phosphorylation sites of HIRA protein. (**d**) Phylogenetic tree derived from homologous amino acid sequence of HIRA.

ProtParam analysis predicted a molecular weight of 111,086.10 Da and an isoelectric point of 8.18. Leucine constituted the most abundant residue (10.5%), while tyrosine was the least frequent (1.4%). TMHMM analysis confirmed the absence of transmembrane domains (Figure 6a). Post-translational modification sites included 1 N-glycosylation site (Asn244) and 114 phosphorylation sites (32 threonine, 3 tyrosine, and 79 serine) (Figure 6b,c).

#### 3.5.3. Phylogenetic Relationships

A phylogenetic tree was created with MEGA X using the Neighbor-Joining method (utilizing 1000 bootstrap replicates) based on HIRA sequences from model organisms (Figure 6d). The phylogenetic tree demonstrated that sequences from domesticated mammalian species (sheep and cattle) clustered separately from avian species and were also distinct from those of common rodent model organisms (rats and mice).

#### 3.5.4. RNA Secondary Structure Modulation via Variants

**Figure 7:**
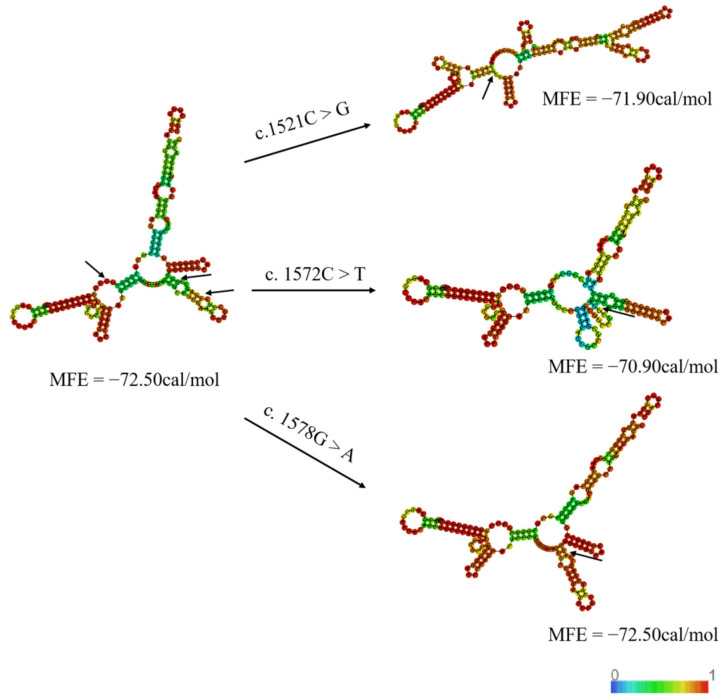
The secondary structure with the wild-type mRNA of exon 14, along with the structural alterations induced by the c.1521C>G, c.1572C>T, and c.1578G>A mutations. The MFE of the secondary structure in exon 14 of the *HIRA* gene was evaluated. MFE predictions were made concerning both the secondary structure and free energy. The structure is colored according to base pairing probabilities, with colors ranging from light to dark representing the likelihood of unpairing.

RNAfold-based minimum free energy (MFE) predictions demonstrated secondary structural alterations in the point mutations [20]. Figure 7 specifically illustrates the protein secondary structural alterations at three litter size-associated variants. The mutations in exon 14 exhibited a decreasing trend in the MFE changes. Specifically, the MFE of c.1521C>T decreased from −72.50 to −71.90 kcal/mol, and that of c.1572C>T decreased from −72.50 to −70.90 kcal/mol. Meanwhile, the MFE of c. 1578G>A did not change. The structural alterations and MFE changes associated with other variants are illustrated in Figure S1.

#### 3.5.5. Structural Consequences of Missense Mutations

**Figure 8:**
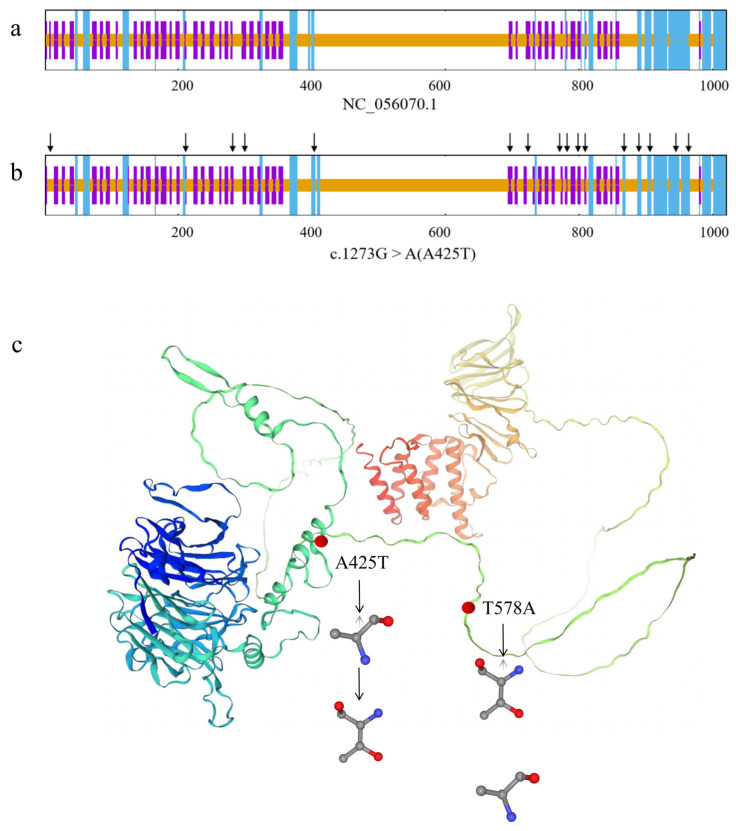
Structural alterations at the secondary and tertiary levels of the ovine HIRA protein. (**a**) The secondary structure of the coding protein for wild-type ovine HIRA. (**b**) The secondary structural modifications changes at c.1273G>A (A425T). The long blue vertical line represents an alpha helix, the purple line denotes an extended strand, and the short yellow line indicates a random coil. The position of the structural change is highlighted by a black arrow. (**c**) The predictions of the tertiary structures of ovine HIRA. The amino acid changes at the A425T (c.1273G>A) and T579A (c.1735A>G) sites are anticipated to result in noticeable alterations in the HIRA structure.

SOPMA analysis predicted secondary structure proportions for wild-type HIRA as follows: 15.00% α-helices, 0.00% β-sheets, 65.20% random coils, and 19.80% extended strands (Figure 8a). The c.1273G>A (Ala425Thr) mutation induced 16 conformational alterations (indicated by black arrows), modifying proportions to 15.29% α-helices, 0.00% β-sheets, 64.22% random coils, and 20.49% extended strands (Figure 8b). Conversely, c.1735A>G (Thr579Ala) showed no secondary structure changes.

Homology modeling using chain A of growth and differentiation factor 8 (PDB ID: AOA667GSB8.1.A; 91.81% sequence identity) demonstrated that both Ala425Thr and Thr579Ala substitutions induced discernible tertiary structural perturbations (Figure 8c).

## 4. Discussion

A growing body of evidence indicates a critical link between the *HIRA* gene and reproduction. Studies conducted by Dilg et al. [8] and Pchelintsev et al. [21] have demonstrated that mutations in this gene significantly increase embryonic mortality rates. In Drosophila models, it has been reported that the overexpression of the dHira protein disrupts the regulation of cell cycle-related genes, thus leading to asynchronous nuclear division and subsequent developmental arrest during early embryogenesis [22]. Interestingly, research has also indicated that while female mice carrying *HIRA* mutations exhibit normal ovulation, they fail to produce offspring when paired with wild-type males [23]. Moreover, Zhou et al. explored genetic associations between specific *HIRA* variants, g.71874104G>A and g.71833755T>C, and litter size in Small-tail Han sheep [14]. In particular, our study is the first study of *HIRA* gene mutations linked to prolificacy in Sonid sheep.

Even though synonymous mutations do not alter the amino acid sequence, they can nonetheless influence the expression, splicing, stability [24,25,26], and secondary structure of mRNA [27,28], in addition to affecting the translation, folding [29], and functional properties of proteins [30]. Synonymous mutations can influence the production of active, properly folded proteins, thereby affecting physiological activity [29]. Research shows that a decrease in MFE may lead to the enhanced stability of the secondary structure of mRNA [30,31]. In our studies, the MFE of the silent LD-Sonid mutation induced a decreasing trend. Rose J et al. reported that even a single base pair alteration can result in changes to the secondary structures of mRNA. [31]. Based on predicted mRNA secondary structural alterations induced by mutations, we postulate that c.1572C>T serves as the primary functional variant due to its significant change in MFE (ΔMFE = −1.6 kcal/mol). Studies have shown that during the maturation of mammalian oocytes, high-stability mRNAs are preferentially retained in post-transcriptional regulation, whereas the accelerated degradation of mRNA during oocyte aging can impair embryonic developmental potential [32]. Increased mRNA stability may prevent the abnormal accumulation of reproduction-related genes, such as maternal mRNAs, thereby supporting normal embryonic development [33]. Enhanced mRNA stability may also promote the spatiotemporal regulation of reproductive genes in germ cells, thus affecting ovarian maturation and oocyte function [34,35]. Furthermore, high-stability mRNAs maintain the stable expression of critical genes under environmental stress, aiding reproductive cells in executing their functions, particularly in response to oxidative stress [36,37]. Recent studies have found that silent SNPs and mRNA secondary structures affect litter size in Awassi and Hamdani sheep [38]. Thus, we propose that c.1572C>T and c.1521C>G could affect the litter size of sheep by enhancing mRNA stability and altering its secondary structure. Although the c.1578G>A mutation did not alter the MFE in our prediction, even single-base changes can induce local structural rearrangements in mRNA that are not fully captured by global MFE calculations [30]. These subtle changes might affect the binding sites for regulatory microRNAs or RNA-binding proteins, thereby influencing HIRA mRNA stability or translation in a tissue-specific manner [39]. Therefore, the combined effect of all three mutations, through a potential interplay of mRNA stability and translation efficiency, might underlie the significant association with litter size. However, further experimental validation is needed to support this hypothesis.

In this study, two missense mutations c.1273G>A (A425T) and c.1735A>G (T425A) were identified. The mutation c.1273G>A exhibited significant departures from HWE, and fewer than ten individuals had the mutation c.1735A>G, which prevented effective association analysis. However, we still conducted analyses of the tertiary structures of the protein to provide references and scientific clues for the study of the *HIRA* gene. The two missense mutations were modeled within the tertiary structure of the HIRA protein, where they were predicted to cause noticeable structural changes. Notably, both missense mutations are localized within the LCD of the HIRA protein, as predicted by SMART analysis. Primarily, these mutations may disrupt the conservation of the amino acid sequence of the LCD, thereby leading to protein conformational disorders through the reduction in protein stability [40]. As a chaperone protein for histone H3.3, a decrease in the stability of HIRA will impair its ability to mediate histone deposition, thereby affecting chromatin remodeling [41]. Crucially, HIRA also regulates the expression of steroidogenic genes, which are controlled by gonadotropins such as Follicle-stimulating hormone receptor (FSHR) and luteinizing hormone/chorionic gonadotropin receptor (LHCGR), ad these also directly modulate estradiol (E_2_) biosynthesis [42,43]. Missense mutations may reduce the chromatin accessibility of these genes and inhibit hormonal synthesis, thereby disrupting the cooperative interaction between FSH/LH and granulosa cells as well as theca cells [44]. Consequently, we postulate that c.1273G>A (Ala425Thr) and c.1735A>G (Thr579Ala) maybe alter LCD architecture to impair granulosa cells functionality, ultimately perturbing oocyte maturation until ovulation in sheep. However, this hypothesis still needs additional experimentation. Future studies should verify the association between mutations and litter size in a large sheep population.

Sonid sheep is a characteristic breed of Mongolia sheep. Recent studies on Mongolia sheep have identified prolificacy-associated mutations in the following key genes: the g.46544883A>G, c.1040T>C, and g.46547859C>T of *GDF9* in Mongolia sheep [18], the c.240C>T and c.279C>T of *LEPR* in both Mongolia and Ujimqin sheep [45], the g.29346567C>T and c.1470G > T of *BMPR1B* in Mongolia sheep [46], and the g.50985975 G>A and c.755 T>C of *BMP15* in Mongolia sheep, along with the g.50988478C>A and g.50987863G>A of *BMP15* in Ujimqin sheep [47]. The identification of breed-specific molecular markers for prolificacy is a key objective in genetic selection programs aimed at improving sheep productivity [48]. Based on the bioinformatic results and association analysis of this study, we identified that the mutations c.1521C>G, c.1572C>T, and c.1578G>A of LD are associated with litter size in Sonid sheep. Hence, the current findings may be utilized in marker-assisted selection (MAS) strategies to enhance prolificacy in low-fertility breeds including Sonid sheep.

## 5. Conclusions

In summary, the results of this study indicated that the LD-Sonid mutation (including c.1521C>G, c.1572C>T, and c.1578G>A) of *HIRA* was associated with litter size in Sonid sheep. These markers could be potentially utilized in MAS to enhance litter size in Sonid sheep. The silent mutation LD-Sonid could potentially improve the stability of mRNA by decreasing the MFE and affecting the secondary structure of *HIRA* mRNA. The missense mutations c.1273G>A (A425T) and c.1735A>G (T425A) induce conformational rearrangements in the tertiary protein structure, potentially altering the chromatin functions of HIRA. The effects of other mutation variants on the litter size of Sonid sheep require further investigation in a larger population of sheep. We should note that the association results require validation in larger, independent cohorts. Furthermore, the functional impact of the identified mutations on mRNA stability and the protein function of HIRA remains to be experimentally validated. In conclusion, these results offer significant genetic markers for sheep breeding and highlight new variants for further functional research on the *HIRA* gene.

## Figures and Tables

**Table 1:** PCR primers used for sequencing *HIRA*.

Primer	Primer Sequence (5′–3′)	Target Region	Annealing Temperature Tm (°C)	Product Size (bp)
HIRA-1	F: CGTGACTTCCGGCGTTGC R: CAGGACCTGCCCCTTCCAG	Exon 1	54	613 (178 bp 5′flanking region + 128 bp exon 1 + 307 bp intron 1)
HIRA-2	F: TCGCTTGCGGCGTTTGTT R: CGCTCGTCCTTCTCGTCATC	Exon 2	56	1071 (120 bp intron 1 + 68 bp exon 2 + 810 bp intron 2 + 73 bp exon 3)
HIRA-3	F: GAGTGGGCTCCTGCTTTAGG R: ATCAGGCGGGAATGGCTC	Exon 3	62	1026 (670 bp intron 2 + 111 bp exon 3 + 245 bp intron 3)
HIRA-4	F: CCCGCAGTAGCGCCATAGT R: GGTGCCAGGGTGGGATAAA	Exon 4	62	782 (618 bp intron 3 + 91 bp exon 4 + 73 bp intron 4)
HIRA-5	F: TTGAGAAACAGAAGCAAATCACAGTCCCACC R: TCCAGACCTGAAAGAGCCCACTGAAGACA	Exon 5	54	740 (311 bp intron 4 + 95 bp exon 5 + 334 bp intron 5)
HIRA-6	F: CTTCAGGGTGGTTTGGACAGT R: CGACAGGGCAGGAACAGGA	Exon 6	62	645 (166 bp intron 5 + 96 bp exon 6 + 383 bp intron 6)
HIRA-7	F: CATTGAGGACGAGCAGGGAC R: CCAACGAGGCAAGGGAGA	Exon 7	54	751 (260 bp intron 6 + 161 bp exon 7 + 330 bp intron 7)
HIRA-8	F: ATAGCAGCATCACCTCACCCT R: AAAGAAGAACCGGACGACAAC	Exon 8	60	790 (467 bp intron 7 + 168 bp exon 8 +155 bp intron 8)
HIRA-9	F: GCAAAGCGTCACCATCTGTT R: ACCTCCAGTCCTGCAACTCC	Exon 9	58	841 (226 bp intron 8 + 114 bp exon 9 + 501 bp intron 9)
HIRA-10/11	F: GTGGTGGGCTGAGCGGACTT R: CAGGCTAACCAAGGAGGGAC	Exon 10/11	62	863 (124 bp intron 9 + 71 bp exon 10 + 207 bp intron 10 + 106 bp exon 11 + 355 bp intron 11)
HIRA-12	F: CCGAGGGTCTCAATTTCAGTT R: TGCAGCCACAAGCACAGG	Exon 12	62	883 (281 bp intron 11 + 231 bp exon 12 + 371 bp intron 12)
HIRA-13	F: TCGGTTATGAGCCTTTGGTG R: CGCTGAGAAAGAGGGTGCTAC	Exon 13	62	710 (341 bp intron 12 + 86 bp exon 13 + 283 bp intron 13)
HIRA-14	F: TCGGGTCTGACCGAATGTAG R: TGTCGCAGAGGCTGTGGTA	Exon 14	62	896 (180 bp intron 13 + 192 bp exon 14 + 524 bp intron 14)
HIRA-15	F: ATGCGCTTCCGGTTAGGC R: GGAGGCGAGGATACGCTGTG	Exon 15	54	634 (321 bp intron 14 + 162 bp exon 15 + 151 bp intron 15)
HIRA-16/17	F: CGTGCTGTTTCCAAAAGGTGTCATCG R: TGCGTGGGAGCAGCCAGAGTCGG	Exon 16/17	62	1008 (403 bp intron 15 + 205 bp exon 16 + 237 bp intron 16 + 105 bp exon 17 + 58 bp intron 17)
HIRA-18	F: CTGACGCCAACCCGCACCAT R: AGAAAGCCTTGCTCACTTCCCT	Exon 18	60	830 (440 bp intron 17 + 149 bp exon 18 + 241 bp intron 18)
HIRA-19/20	F: ACCACGGGTGCTCACTG R: CCTGGACTATGGCTAAGGGT	Exon 19/20	60	948 (143 bp intron 18 + 221 bp exon 19 +154 bp intron 19 + 106 bp exon 20 + 324 bp intron 20)
HIRA-21/22	F: CTACTGCTTCAGCCCGTCATTG R: CCCAGCCTTTCCGCTCACA	Exon 21/22	60	796 (244 bp intron 20 + 123 bp exon 21 + 178 bp intron 21 + 164 bp exon 22 + 87 bp intron 22)
HIRA-23	F: TGCGTTTGTGGTAACTTGGC R: AGGAAGGCGGAAGGCTCA	Exon 23	62	719 (78 bp intron 22 + 89 bp exon 23 + 552 bp intron 23)
HIRA-24-1	F: CCTTCCTTTATAGGTACGATGTGCCTAATCTTTGG R: TCCTTGGGCTGGGACAGGCTTTCCTTTTA	Exon 24	54	1434 (919 bp intron 23 + 515 bp exon 24)
HIRA-24-2	F: CGGTGGATGAGCCATTGTG R: GCCTGCCGCCTAAGAAAA	Exon 24	62	669 (378 bp exon 24 + 291 bp 3′ untranslated region)

Note: F—forward primer; R—reverse primer.

**Table 2:** The effect of the genotypes of the variants in *HIRA* on litter size in the Sonid sheep population.

Variant	Genotype	Number	Litter Size
c.612G>A	GG	356	1.14 ± 0.02
	GA	28	1.07 ± 0.05
c.1521C>G in LD-Sonid	CC	340	1.07 ± 0.01 ^A^
	CG	42	1.64 ± 0.07 ^B^
c.1941G>A	GG	142	1.18 ± 0.03
	GA	167	1.14 ± 0.03
	AA	74	1.05 ± 0.03
c.2682C>T	CC	349	1.13 ± 0.02
	CT	33	1.33 ± 0.06
c.3449C>G	CC	320	1.14 ± 0.02
	CG	60	1.13 ± 0.04

Note: Different uppercase letters in the same group indicate a significant difference ^A, B^: *p* < 0.01. The mutations c.1572C>T and c.1578G>A are not listed separately as they show in complete LD with c.1521C>G (collectively referred to as LD-Sonid), and thus, their genotypic distributions and effects on litter size are identical to those shown for c.1521C>G.

## Data Availability

The original contributions presented in this study are included in the article/Supplementary Materials. Further inquiries can be directed to the corresponding author.

## Supplementary Materials

The following supporting information can be downloaded at: https://www.mdpi.com/article/10.3390/ani15202936/s1, Supplementary Table S1. MassARRAY primers used for genotyping of 15 variants in *HIRA* gene. Supplementary Table S2. Genotypic and allelic frequencies and diversity parameters of each variant in Sonid sheep. Supplementary Table S3. Linkage disequilibrium as measured by D’ and *r*^2^ among variants in Sonid sheep. Supplementary Figure S1. The secondary structure with the wild-type mRNA and the secondary structural changes caused by mutations. The minimum free energy of the secondary structure of the *HIRA* gene. MFE prediction in terms of the secondary structure and free energy.

## References

[1] China National Commission of Animal Genetic Resources (CNCAGR) (2011). Sheep and Goats, Animal Genetic Resources in China.

[2] Xiang J., Li H., Guo Z., Li T., Yamada T., Li X., Bao S., Da L., Borjigin G., Cang M. (2025). Effect of *FABP4* Gene Polymorphisms on Fatty Acid Composition, Chemical Composition, and Carcass Traits in Sonid Sheep. Animals.

[3] Paz E., Quiñones J., Bravo S., Montaldo H.H., Sepúlveda N. (2015). Genotyping of *BMPR1B*, *BMP15* and *GDF9* genes in Chilean sheep breeds and association with prolificacy. Anim. Genet..

[4] Ahlawat S., Sharma R., Roy M., Mandakmale S., Prakash V., Tantia M.S. (2016). Genotyping of Novel SNPs in *BMPR1B, BMP15*, and *GDF9* Genes for Association with Prolificacy in Seven Indian Goat Breeds. Anim. Biotechnol..

[5] Liu Q.Y., Pan Z.Y., Wang X.Y., Hu W.P., Di R., Yao Y.X. (2014). Progress on major genes for high fecundity in ewes. Front. Agric. Sci. Eng..

[6] Rai T.S., Puri A., McBryan T., Hoffman J., Tang Y., Pchelintsev N.A., van Tuyn J., Marmorstein R., Schultz D.C., Adams P.D. (2011). Human CABIN1 is a functional member of the human HIRA/UBN1/ASF1a histone H3.3 chaperone complex. Mol. Cell Biol..

[7] Yang J.H., Song T.Y., Jo C., Park J., Lee H.Y., Song I. (2016). Differential regulation of the histone chaperone HIRA during muscle cell differentiation by a phosphorylation switch. Exp. Mol. Med..

[8] Dilg D., Saleh R.N., Phelps S.E., Rose Y., Dupays L., Murphy C. (2016). HIRA Is Required for Heart Development and Directly Regulates Tnni2 and Tnnt3. PLoS ONE.

[9] Ray-Gallet D., Quivy J.P., Scamps C., Martini E.M., Lipinski M., Almouzni G. (2002). HIRA is critical for a nucleosome assembly pathway independent of DNA synthesis. Mol. Cell.

[10] van der Heijden G.W., Dieker J.W., Derijck A.A., Muller S., Berden J.H., Braat D.D. (2005). Asymmetry in histone H3 variants and lysine methylation between paternal and maternal chromatin of the early mouse zygote. Mech. Dev..

[11] Horard B., Sapey-Triomphe L., Bonnefoy E., Loppin B. (2018). ASF1 is required to load histones on the HIRA complex in preparation of paternal chromatin assembly at fertilization. Epigenetics Chromatin.

[12] Roberts C., Sutherland H.F., Farmer H., Kimber W., Halford S., Carey A. (2002). Targeted mutagenesis of the *Hira* gene results in gastrulation defects and patterning abnormalities of mesoendodermal derivatives prior to early embryonic lethality. Mol. Cell Biol..

[13] Nashun B., Hill P.W., Smallwood S.A., Dharmalingam G., Amouroux R., Clark S.J. (2015). Continuous Histone Replacement by Hira Is Essential for Normal Transcriptional Regulation and De Novo DNA Methylation during Mouse Oogenesis. Mol. Cell.

[14] Zhou M., Pan Z., Cao X., Guo X., He X., Sun Q. (2018). Single Nucleotide Polymorphisms in the *HIRA* Gene Affect Litter Size in Small Tail Han Sheep. Animals.

[15] Shah R., Sharma V., Bhat A., Singh H., Sharma I., Verma S., Bhat G.R., Sharma B., Bakshi D., Kumar R. (2020). MassARRAY analysis of twelve cancer related SNPs in esophageal squamous cell carcinoma in J&K, India. BMC Cancer.

[16] Masatoshi N., Roychoudhury A.K. (1974). Sampling variances of heterozygosity and genetic distance. Genetics.

[17] Barrett J.C., Fry B., Maller J., Daly M.J. (2005). Haploview: Analysis and visualization of LD and haplotype maps. Bioinformatics.

[18] Tong B., Wang J.P., Cheng Z.X., Liu J.S., Wu Y.R., Li Y.H. (2020). Novel variants in *GDF9* gene affect promoter activity and litter size in Mongolia sheep. Genes.

[19] Sweet R.M., Eisenberg D. (1983). Correlation of sequence hydrophobicities measures similarity in three-dimensional protein structure. J. Mol. Biol..

[20] Mathews D.H., Disney M.D., Childs J.L., Schroeder S.J., Michael Z., Turner D.H. (2004). Incorporating chemical modification con-straints into a dynamic programming algorithm for prediction of RNA secondary structure. Proc. Natl. Acad. Sci. USA.

[21] Pchelintsev N.A., McBryan T., Rai T.S., van Tuyn J., Ray-Gallet D., Almouzni G. (2013). Placing the HIRA histone chaperone com-plex in the chromatin landscape. Cell Rep..

[22] Wang Y.F., Xu Y.J. (2005). Effects of *Hira* gene overexpression on early embryonic development in Drosophila melanogaster. J. Cent. China Norm. Univ. (Nat. Sci. Ed.).

[23] Lin C.J., Koh F.M., Wong P., Conti M., Ramalho-Santos M. (2014). Hira-mediated H3.3 incorporation is required for DNA replication and ribosomal RNA transcription in the mouse zygote. Dev. Cell.

[24] Franco P., Michela R., Baralle F.E. (2005). Synonymous mutations in CFTR exon 12 affect splicing and are not neutral in evolution. Proc. Natl. Acad. Sci. USA.

[25] Duan J., Wainwright M.S., Comeron J.M., Saitou N., Sanders A.R., Gelernter J. (2003). Synonymous mutations in the human do-pamine receptor D2 (DRD2) affect mRNA stability and synthesis of the receptor. Hum. Mol. Genet..

[26] Chava K.S., Jung M.O., Kim I.W., Sauna Z.E., Anna M.C., Ambudkar S.V. (2007). A “silent” polymorphism in the *MDR1* gene changes substrate specificity. Science.

[27] Mohan G.A. (2018). A-Adducin nsSNPs affect mRNA secondary structure, protein modification and stability. Meta Gene.

[28] Rachel S., Cygan K.J., Rhine C.L., Glidden D.T., Taggart A.J., Lin C.L. (2017). The effects of structure on pre-mRNA processing and stability. Methods.

[29] Sauna Z.E., Kimchi-Sarfaty C. (2011). Understanding the contribution of synonymous mutations to human disease. Nat. Rev. Genet..

[30] Imran F.S., Al-Thuwaini T.M. (2024). The novel C268A variant of *BMP2* is linked to the reproductive performance of Awassi and Hamdani sheep. Mol. Biol. Rep..

[31] Rose J., Kraft T., Brenner B., Montag J. (2020). Hypertrophic cardiomyopathy *MYH7* mutation R723G alters mRNA secondary structure. Physiol. Genom..

[32] Kong L., Gong Y., Wang Y., Yuan M., Liu W., Zhou H. (2025). Multi-omics revealed that DCP1A and SPDL1 determine embryo-genesis defects in postovulatory ageing oocytes. Cell Prolif..

[33] Sha Q.Q., Zheng W., Wu Y.W., Li S., Guo L., Zhang S. (2020). Dynamics and clinical relevance of maternal mRNA clearance during the oocyte-to-embryo transition in humans. Nat. Commun..

[34] Peng F., Nordgren C.E., Murray J.I. (2024). A spatiotemporally resolved atlas of mRNA decay in the C. elegans embryo reveals differential regulation of mRNA stability across stages and cell types. Genome Res..

[35] Niu X., Huang Y., Lu H., Li S., Huang S., Ran X. (2022). CircRNAs in Xiang pig ovaries among diestrus and estrus stages. Porc. Health Manag..

[36] Shim J.S., Park S.H., Lee D.K., Kim Y.S., Park S.C., Redillas M.C.F.R. (2021). The Rice GLYCINE-RICH PROTEIN 3 Confers Drought Tolerance by Regulating mRNA Stability of ROS Scavenging-Related Genes. Rice.

[37] Geisberg J.V., Moqtaderi Z., Struhl K. (2023). Condition-specific 3′ mRNA isoform half-lives and stability elements in yeast. Proc. Natl. Acad. Sci. USA.

[38] Froebel B.R., Trujillo A.J., Sullivan J.M. (2017). Effects of Pathogenic Variations in the Human Rhodopsin Gene (hRHO) on the Predicted Accessibility for a Lead Candidate Ribozyme. Investig. Ophthalmol. Vis. Sci..

[39] Zeng X., Wei Z., Du Q., Li J., Xie Z., Wang X. (2024). Unveil cis-acting combinatorial mRNA motifs by interpreting deep neural network. Bioinformatics.

[40] Beltran A., Jiang X., Shen Y., Lehner B. (2025). Site-saturation mutagenesis of 500 human protein domains. Nature.

[41] Zhu J., Chen K., Sun Y.H., Ye W., Liu J., Zhang D. (2023). LSM1-mediated Major Satellite RNA decay is required for nonequilibrium histone H3.3 incorporation into parental pronuclei. Nat. Commun..

[42] Samie K.A., Kowalewski M.P., Schuler G., Gastal G.D.A., Bollwein H., Scarlet D. (2025). Roles of *GDF9* and *BMP15* in equine fol-licular development: In vivo content and in vitro effects of IGF1 and cortisol on granulosa cells. BMC Vet. Res..

[43] Abedal-Majed M.A., Abuajamieh M., Al-Qaisi M., Sargent K.M., Titi H.H., Alnimer M.A. (2023). Sheep with ovarian androgen excess have fibrosis and follicular arrest with increased mRNA abundance for steroidogenic enzymes and gonadotropin receptors. J. Anim. Sci..

[44] Morozov V.M., Riva A., Sarwar S., Kim W.J., Li J., Zhou L. (2023). HIRA-mediated loading of histone variant H3.3 controls andro-gen-induced transcription by regulation of AR/BRD4 complex assembly at enhancers. Nucleic Acids Res..

[45] Ma S.J., Ji X.W., Cang M., Wang J.G., Yu H.Q., Liu Y.B. (2022). Association analysis between novel variants in *LEPR* gene and litter size in Mongolia and Ujimqin sheep breeds. Theriogenology.

[46] Gao Y.Y., Hao Q., Cang M., Wang J.G., Yu H.Q., Liu Y.B. (2021). Association between novel variants in *BMPR1B* gene and litter size in Mongolia and Ujimqin sheep breeds. Reprod. Domest. Anim..

[47] Wang Y., Chi Z., Jia S., Zhao S., Cao G., Purev C. (2023). Effects of novel variants in *BMP15* gene on litter size in Mongolia and Ujimqin sheep breeds. Theriogenology.

[48] Mura M.C., Cosso G., Ouadday M., Hosri C., Starič J., Nehme M. (2025). Identifying Key Genetic Factors Influencing Reproductive Performance in Dairy and Meat Sheep Breeds of the Mediterranean Region. Reprod. Domest. Anim..

