# Supplementary material for: Novel Silent Mutations in the HIRA Gene Associated with Litter Size in Sonid Sheep
Source: Animals (Basel). 2025 Oct 10;15(20):2936. doi: 10.3390/ani15202936 (PMC12560918; doi:10.3390/ani15202936)
Supplement: Supplementary file 1 [file animals-15-02936-s001.zip › Supplementary Figure S1.pdf]

Supplementary Figure S1

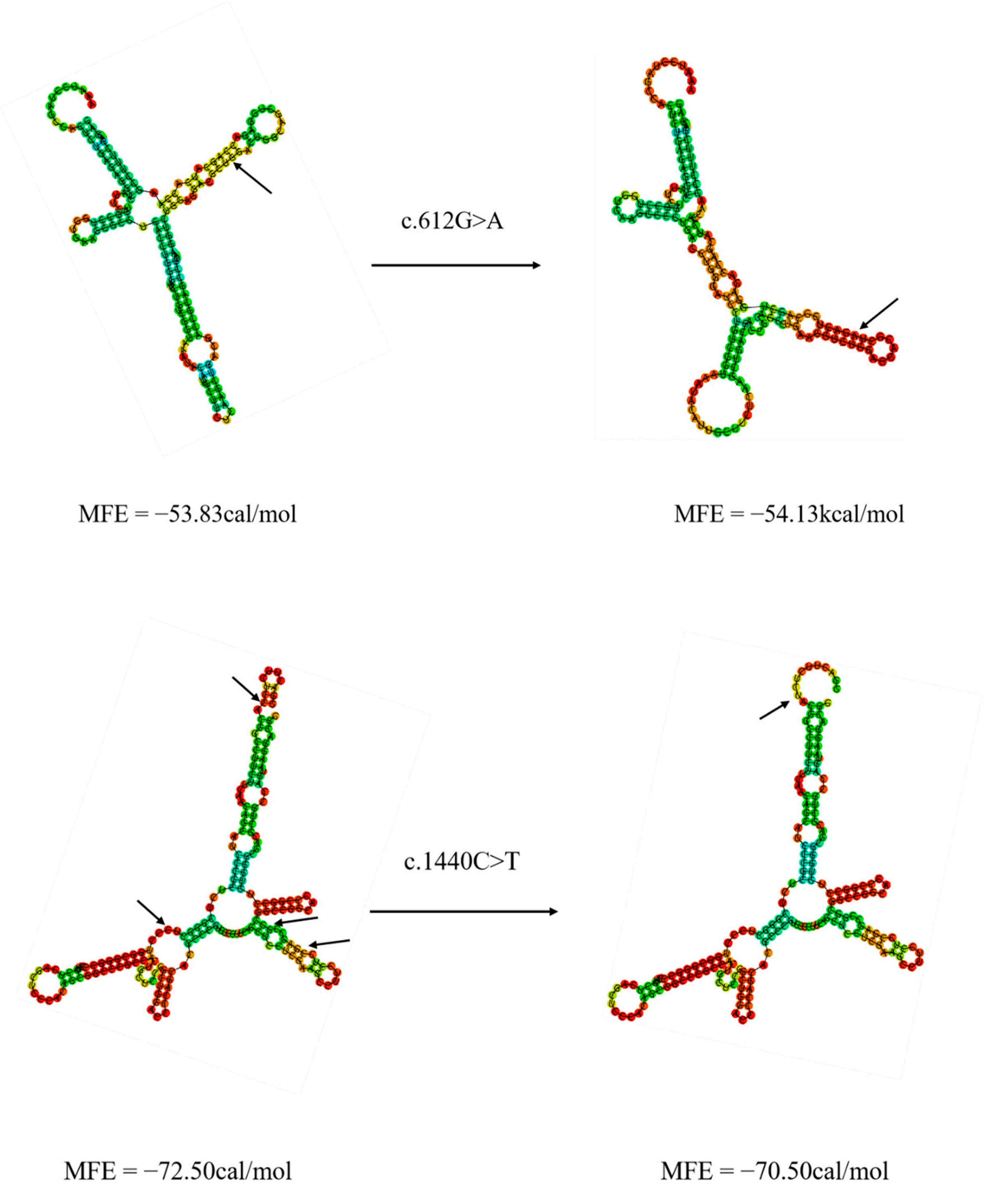

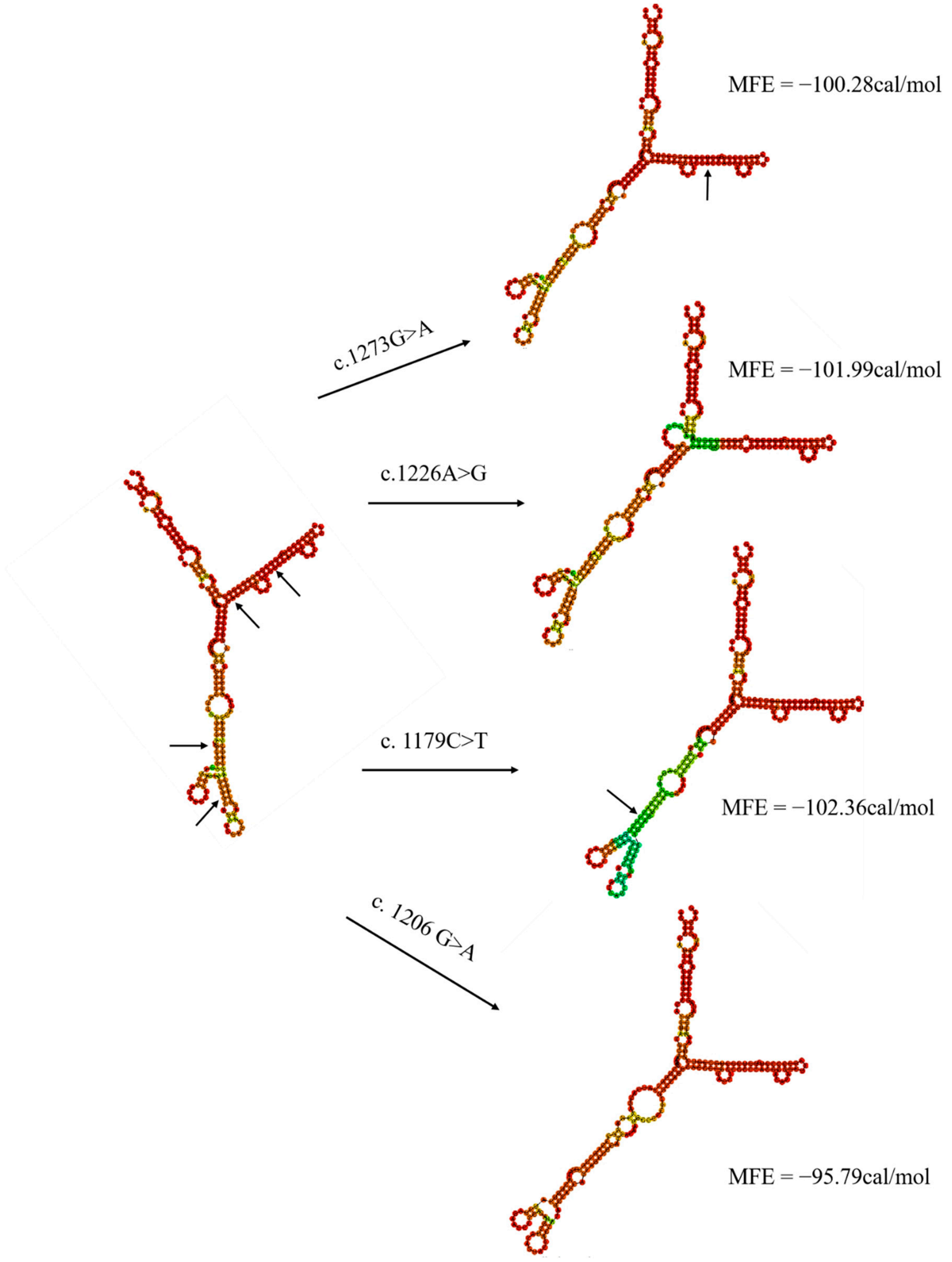
MFE = −100.28cal/mol
c.1273G>A
MFE = −101.99cal/mol
c.1226A>G
c. 1179C>T
MFE = −102.36cal/mol
c. 1206 G>A
MFE = −95.79cal/mol

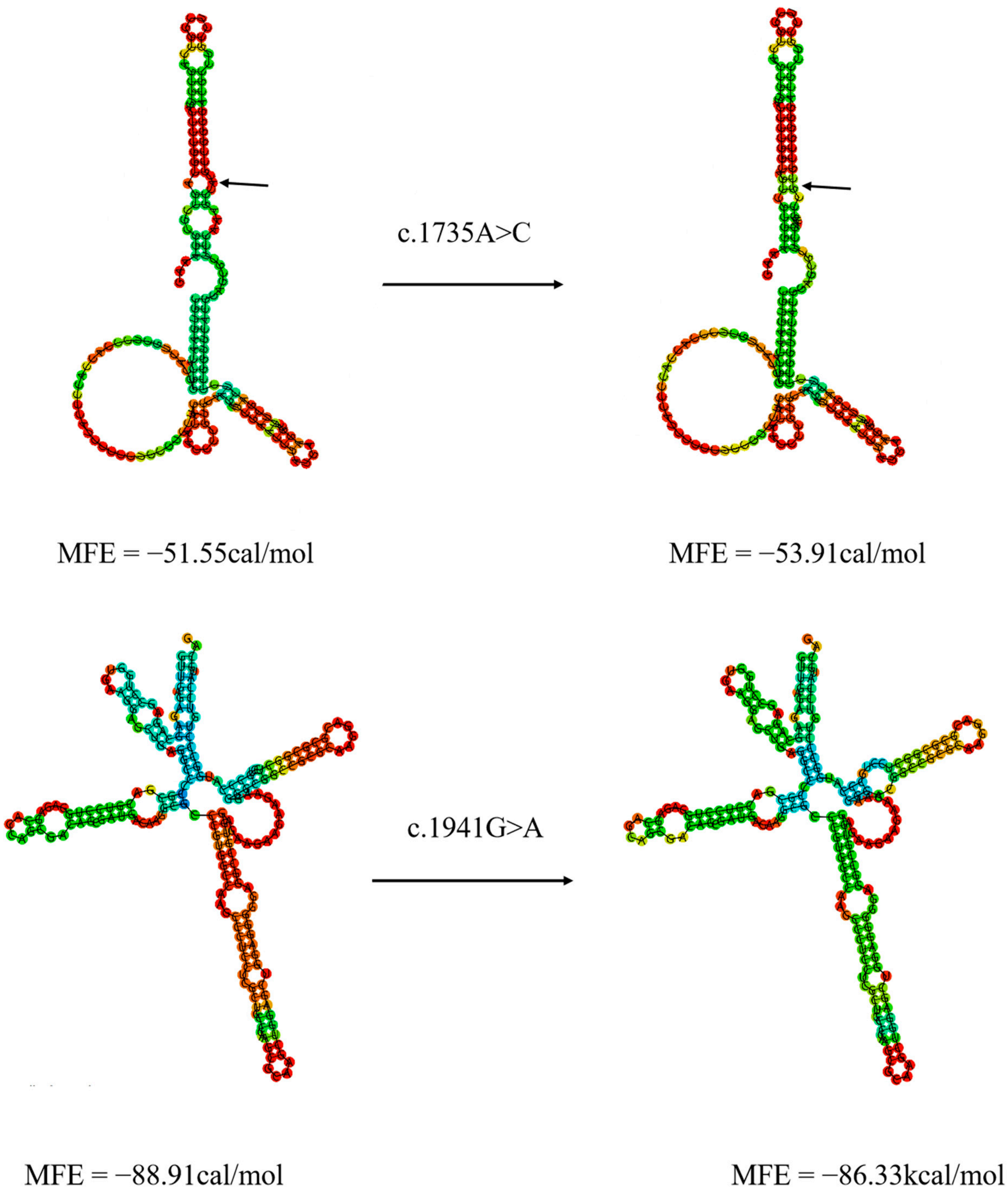
c.1735A>C
MFE = −51.55cal/mol
MFE = −53.91cal/mol
c.1941G>A
MFE = −88.91cal/mol
MFE = −86.33kcal/mol

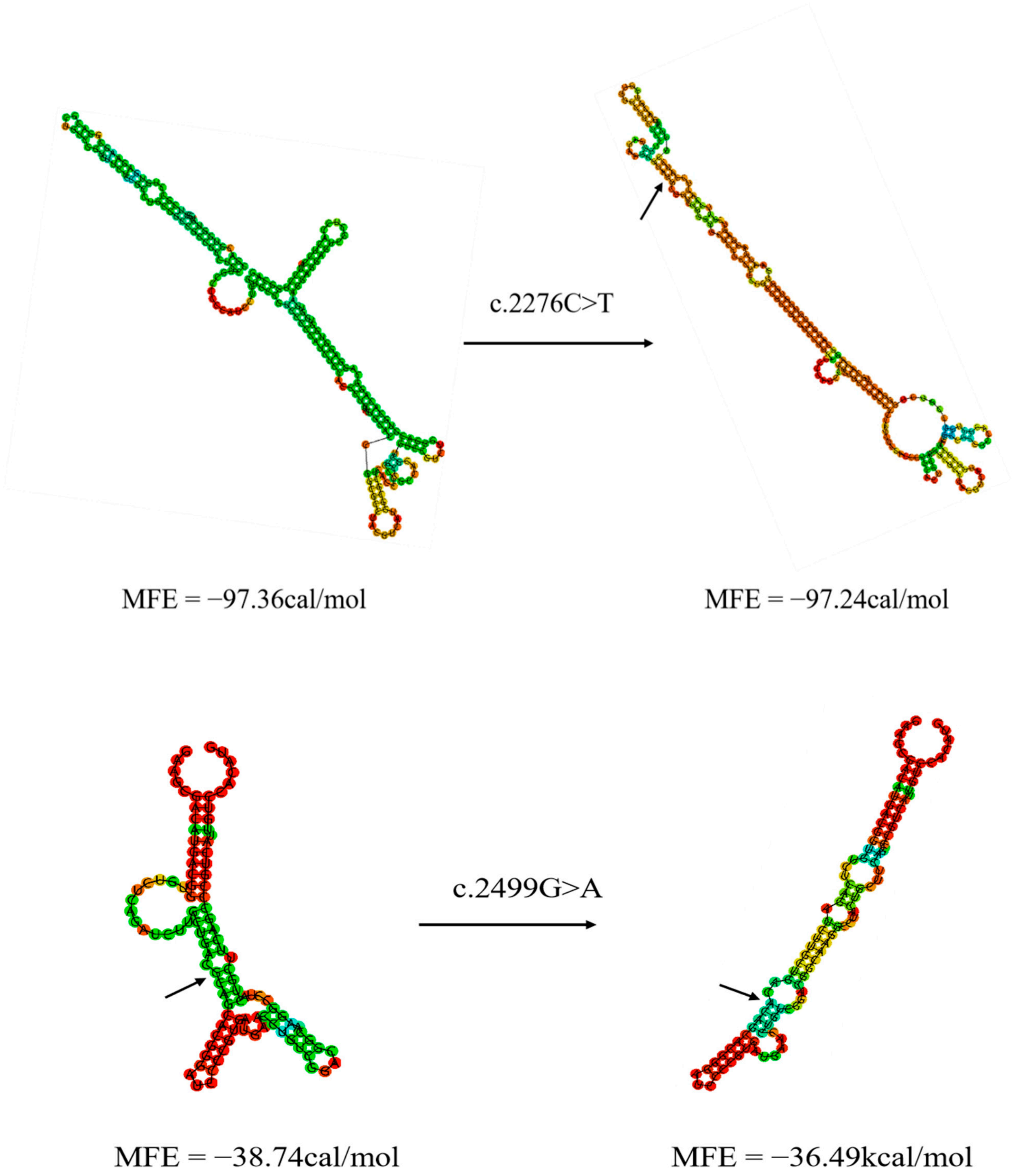
c.2276C>T
MFE = −97.36cal/mol
MFE = −97.24cal/mol
c.2499G>A
MFE = −38.74cal/mol
MFE = −36.49kcal/mol

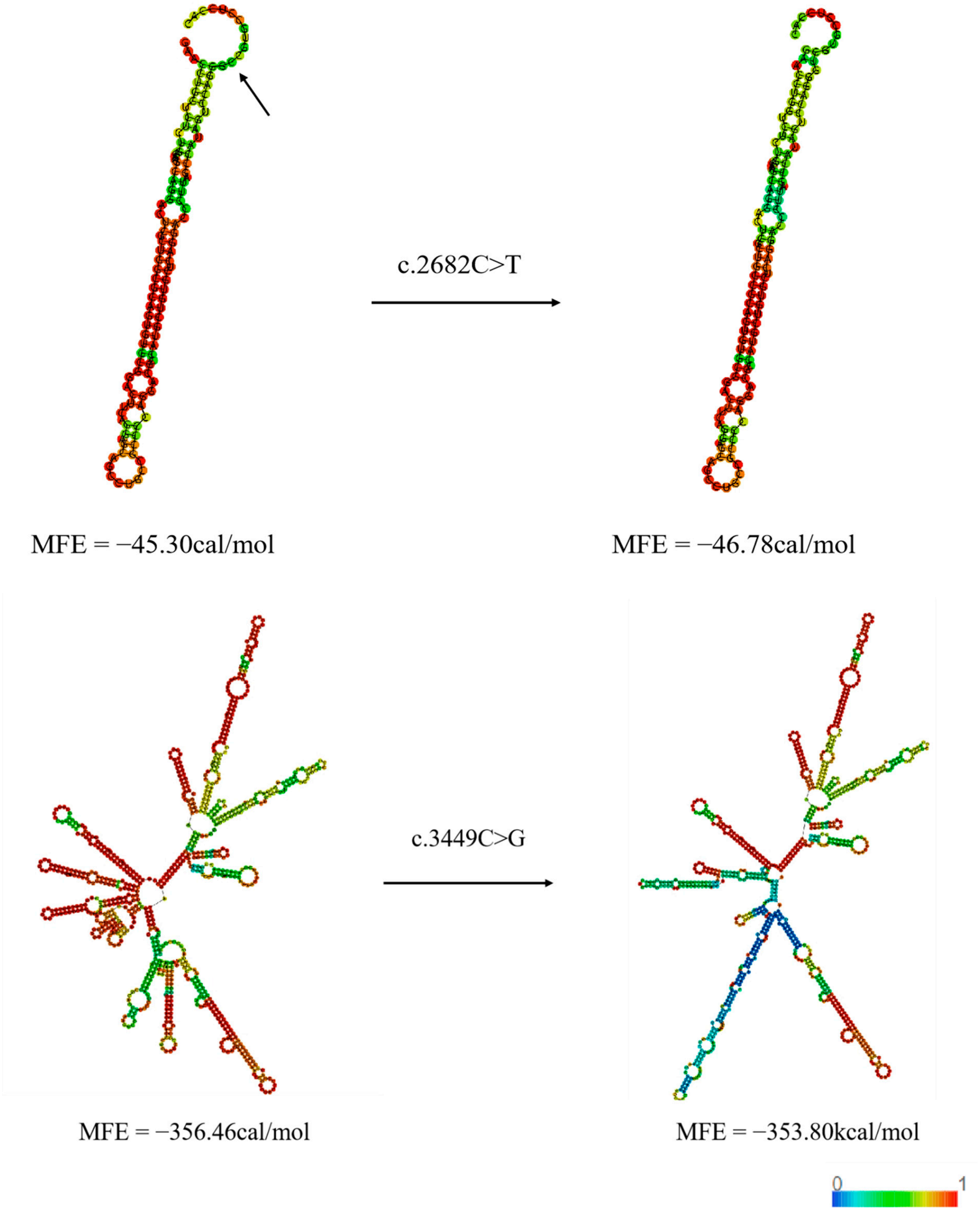

Figure. S1. The secondary structure with the wild-type mRNA of exons and the secondary structural changes caused by variants. MFE of secondary structure in exons of the *HIRA* gene. MFE prediction in terms of the secondary structure and free energy. The structure above is colored according to base pairing probabilities. Colors ranging from light to dark indicate the probability of unpairing.
