# Supplementary material for: Novel Silent Mutations in the HIRA Gene Associated with Litter Size in Sonid Sheep
Source: Animals (Basel). 2025 Oct 10;15(20):2936. doi: 10.3390/ani15202936 (PMC12560918; doi:10.3390/ani15202936)
Supplement: Supplementary file 1 [file animals-15-02936-s001.zip › Supplementary Table S1.pdf]

Supplementary Table S1. MassARRAY primers used for genotyping of sixteen variants in *HIRA* gene.

Name	Target Region	Primer Sequence (5'-3')	Annealing Temperature (°C)
c.612G>A	Exon 7	F: ACGTTGGATGAAGGCTTGGTGATGCTGGTC	49.1
		R: ACGTTGGATGGGGATCCTGTCGGTAAATAC	
		E: CTCCAGCTGCCAGTC	
c.1179C>T	Exon 12	F: ACGTTGGATGAAGAGCCTGGCCATCATGAC	55.6
		R: ACGTTGGATGATTGACGACGCCAGCGACC	
		E: AGAGGCCCAGCTGTCCAC	
c.1206G>A	Exon 12	F: ACGTTGGATGATTGACGACGCCAGCGACC	53.1
		R: ACGTTGGATGAAGAGCCTGGCCATCATGAC	
		E: CTGCCTGTGCTGGTACTG	
c.1226A>G	Exon 12	F: ACGTTGGATGAAGAGCCTGGCCATCATGAC	62.3
		R: ACGTTGGATGATTGACGACGCCAGCGACC	
		E: CCTCGGCCAGGGACGCCGC	
c.1273G>A	Exon 12	F: ACGTTGGATGATTGACGACGCCAGCGACC	57.8
		R: ACGTTGGATGAAGAGCCTGGCCATCATGAC	
		E: CTTCGGAGCCTGTGTCCCTGG	
c.1440C>T	Exon 14	F: ACGTTGGATGTGGGCTGACTCTCCCTTTCT	49.1
		R: ACGTTGGATGAGAGCGGGATGCTGTTGAAG	
		E: GCGACAGGGACTTCTC	
c.1521C>G	Exon 14	F: ACGTTGGATGGACTCACCCGTCCTTACTG	65.9
		R: ACGTTGGATGATGCTCAGCTCCCACAGCG	
		E: CTGGCGTGCTGGGGTCCAGCGG	
c.1572C>T	Exon 14	F: ACGTTGGATGATGCTCAGCTCCCACAGCG	45.3
		R: ACGTTGGATGGACTCACCCGTCCTTACTG	
		E: CTCGAAGCCTTCCTC	

c. 1578G>A	Exon 14	F: ACGTTGGATGGACTCACCCGTCCTTACTG	73.4
		R: ACGTTGGATGATGCTCAGCTCCCACAGCG	
		E: CCCACAGGCCGGGTGCCGGCCGC	
c.1735A>G	Exon 15	F: ACGTTGGATGAACCTATGAAGGCCTTCGAC	59.2
		R: ACGTTGGATGAGCCACCACCTACCTTTCC	
		E: TGCACGGAGCGCTCCAAAGCC	
c.1941G>A	Exon 16	F: ACGTTGGATGAAGCGCAAGCTGGAGCTGGA	48.8
		R: ACGTTGGATGCTCACCTGCATGGACAGGG	
		E: TGGAGAAGAAGAAGAAGGG	
c.2276C>T	Exon 19	F: ACGTTGGATGGTGTGCGTCGCCTGTGAGA	51.9
		R: ACGTTGGATGTGCAGTGCAGGGTGGAGATG	
		E: GTGCCTGTGAGAAGCGGA	
c.2499G>A	Exon 20	F: ACGTTGGATGTGGACAATGACGGGCTGAAG	58.3
		R: ACGTTGGATGAAGCGACATGACGGTGTCTC	
		E: CCCCACGGGGATCCCGTGCTG	
c.2682C>T	Exon 21	F: ACGTTGGATGCTCAGGACCCTTAGCCATAG	48.9
		R: ACGTTGGATGAGGGACCGCATGTGCACAG	
		E: CACTTAGCCATAGTCCAGGG	
c.3449C>G	Exon 24	F: ACGTTGGATGTCAGTAGACTGACCTGAGAG	47.4
		R: ACGTTGGATGTGGCCATTTTACAAGGTAGC	
		E: ATGTAAAAGGAAAGCCTGT	

Note: F: forward primer sequence, R: reverse primer sequence, E: extended primer sequence.
