# Supplementary material for: Novel Silent Mutations in the HIRA Gene Associated with Litter Size in Sonid Sheep
Source: Animals (Basel). 2025 Oct 10;15(20):2936. doi: 10.3390/ani15202936 (PMC12560918; doi:10.3390/ani15202936)
Supplement: Supplementary file 1 [file animals-15-02936-s001.zip › Supplementary Table S2.pdf]

Supplementary Table S2. Genotypic, allelic frequencies and diversity parameters of ten SNPs in Sonid sheep.

SNPs	Genotypic Frequencies			Allelic Frequencies		Diversity Parameters				
						Ho	He	n_e	PIC [1]	χ^2 (HWE[2]) [2]
c.612G>A	GG	GA	AA	G	A					
	0.927	0.073	0.000	0.964	0.036	0.930	0.070	1.076	0.067	0.550 ($p > 0.05$)
c.1179C>T	CC	CT	TT	C	T					
	0.269	0.156	0.575	0.347	0.653	0.547	0.453	1.829	0.350	163.333 ($p < 0.01$)
c.1206G>A	GG	GA	AA	G	A					
	0.369	0.053	0.578	0.396	0.604	0.522	0.478	1.917	0.364	299.980 ($p < 0.01$)
c.1226A>G	AA	AG	GG	A	G					
	0.583	0.000	0.417	0.583	0.417	0.514	0.486	1.946	0.368	379.000 ($p < 0.01$)
c.1273G>A	GG	GA	AA	G	A					
	1.000	0.000	0.000	1.000	0.000	1.000	0.000	1.000	0.000	
c.1440C>T	CC	CT	TT	C	T					
	0.990	0.010	0.000	0.995	0.005	0.990	0.010	1.010	0.010	0.011 ($p > 0.05$)
c.1521C>G	CC	CG	GG	C	G					
	0.885	0.110	0.005	0.940	0.060	0.887	0.113	1.128	0.106	0.309 ($p > 0.05$)
c.1572C>T	CC	CT	TT	C	T					
	0.897	0.103	0.000	0.949	0.051	0.903	0.097	1.108	0.092	1.112 ($p > 0.05$)
c.1578G>A	GG	GA	AA	G	A					
	0.893	0.102	0.005	0.944	0.056	0.894	0.106	1.119	0.100	0.585 ($p > 0.05$)
c.1735A>G	AA	AG	GG	A	G					
	0.995	0.005	0.000	0.997	0.003	0.995	0.005	1.005	0.006	0.003 ($p > 0.05$)
c.1941G>A	GG	GA	AA	G	A					
	0.371	0.436	0.193	0.589	0.411	0.516	0.484	1.939	0.367	3.796 ($p > 0.05$)
c.2276C>T	CC	CT	TT	C	T					

	0.992	0.008	0.000	0.996	0.004	0.992	0.008	1.008	0.008	0.006 ($p > 0.05$)
c.2499G>A	GG	GA	AA	G	A					
	0.466	0.393	0.141	0.663	0.337	0.553	0.447	1.808	0.006	5.560 ($p < 0.05$)
c.2682C>T	CC	CT	TT	C	T					
	0.911	0.086	0.003	0.954	0.046	0.913	0.087	1.096	0.084	0.055 ($p > 0.05$)
c.3449C>G	CC	CG	GG	C	G					
	0.831	0.158	0.010	0.910	0.090	0.837	0.163	1.195	0.150	0.322 ($p > 0.05$)

Note: H_o: observed heterozygosity; H_e: expected heterozygosity; n_e: effective allele numbers; PIC: polymorphism information content; HWE: Hardy-Weinberg equilibrium; 1: The classification was conducted according to the PIC value (PIC value < 0.25, low polymorphism; $0.25 <$ PIC value < 0.5, moderate polymorphism; and PIC value > 0.5, high polymorphism); 2: There were Hardy-Weinberg deviation from the obtained genotype frequency.
