# Supplementary material for: Novel Silent Mutations in the HIRA Gene Associated with Litter Size in Sonid Sheep
Source: Animals (Basel). 2025 Oct 10;15(20):2936. doi: 10.3390/ani15202936 (PMC12560918; doi:10.3390/ani15202936)
Supplement: Supplementary file 1 [file animals-15-02936-s001.zip › Supplementary Table S3.pdf]

Supplementary Table S3. Linkage disequilibrium as measured by D' and r^2 among variants in Sonid sheep.

Variants	c.612G>A	c.1440C>T	c.1521C>G	c.1572C>T	c.1578G>A	c.1735A>G	c.1941G>A	c.2276C>T	c.2682C>T
c.1440C > T	D' = 1.000/ r^2 = 0.000								
c.1521C > G	D' = 0.904/ r^2 = 0.002	D' = 0.433/ r^2 = 0.015							
c.1572C > T	D' = 0.810/ r^2 = 0.001	D' = 1.000/ r^2 = 0.000	D' = 1.000/ r^2 = 0.953						
c.1578G > A	D' = 0.840/ r^2 = 0.002	D' = 1.000/ r^2 = 0.000	D' = 1.000/ r^2 = 0.954	D' = 1.000/ r^2 = 1.000					
c.1735A > G	D' = 0.461/ r^2 = 0.015	D' = 1.000/ r^2 = 0.000	D' = 1.000/ r^2 = 0.000	D' = 1.000/ r^2 = 0.000	D' = 1.000/ r^2 = 0.000				
c.1941G > A	D' = 1.000/ r^2 = 0.042	D' = 1.000/ r^2 = 0.004	D' = 1.000/ r^2 = 0.045	D' = 1.000/ r^2 = 0.042	D' = 1.000/ r^2 = 0043	D' = 1.000/ r^2 = 0.002			
c.2276C > T	D' = 0.282/ r^2 = 0.008	D' = 1.000/ r^2 = 0.000	D' = 0.243/ r^2 = 0.004	D' = 0.250/ r^2 = 0.004	D' = 0.248/ r^2 = 0.004	D' = 1.000/ r^2 = 0.000	D' = 1.000/ r^2 = 0.003		
c.2682C > T	D' = 1.000/ r^2 = 0.002	D' = 1.000/ r^2 = 0.000	D' = 1.000/ r^2 = 0.003	D' = 1.000/ r^2 = 0.003	D' = 1.000/ r^2 = 0.003	D' = 1.000/ r^2 = 0.000	D' = 0.810/ r^2 = 0.022	D' = 1.000/ r^2 = 0.000	
c.3449C > G	D' = 0.002/ r^2 = 0.000	D' = 1.000/ r^2 = 0.001	D' = 1.000/ r^2 = 0.006	D' = 1.000/ r^2 = 0.006	D' = 1.000/ r^2 = 0.006	D' = 1.000/ r^2 = 0.000	D' = 1.000/ r^2 = 0.068	D' = 1.000/ r^2 = 0.040	D' = 0.967/ r^2 = 0.460
